# Potential Role of Deer Tick Virus in Powassan Encephalitis Cases in Lyme Disease–endemic Areas of New York, USA

**DOI:** 10.3201/eid1912.130903

**Published:** 2013-12

**Authors:** Marc Y. El Khoury, Jose F. Camargo, Jennifer L. White, Bryon P. Backenson, Alan P. Dupuis, Kay L. Escuyer, Laura Kramer, Kirsten St. George, Debarati Chatterjee, Melissa Prusinski, Gary P. Wormser, Susan J. Wong

**Affiliations:** New York Medical College, Valhalla, NY, USA (M.Y. El Khoury, J.F. Camargo, D. Chatterjee, G.P. Wormser);; Wadsworth Center, New York State Department of Health, Albany, NY, USA (J.L. White, B.P. Backenson, A.P. Dupuis II, K.L. Escuyer, L. Kramer, K. St. George, M. Prusinski, S.J. Wong)

**Keywords:** deer tick virus, Powassan virus, encephalitis, flavivirus, Ixodes scapularis, New York, United States, ticks, vector-borne infections, viruses, Lyme disease

## Abstract

Powassan virus, a member of the tick-borne encephalitis group of flaviviruses, encompasses 2 lineages with separate enzootic cycles. The prototype lineage of Powassan virus (POWV) is principally maintained between *Ixodes cookei* ticks and the groundhog (*Marmota momax*) or striped skunk (*Mephitis mephitis*), whereas the deer tick virus (DTV) lineage is believed to be maintained between *Ixodes scapularis* ticks and the white-footed mouse (*Peromyscus leucopus*). We report 14 cases of Powassan encephalitis from New York during 2004–2012. Ten (72%) of the patients were residents of the Lower Hudson Valley, a Lyme disease–endemic area in which *I. scapularis* ticks account for most human tick bites. This finding suggests that many of these cases were caused by DTV rather than POWV. In 2 patients, DTV infection was confirmed by genetic sequencing. As molecular testing becomes increasingly available, more cases of Powassan encephalitis may be determined to be attributable to the DTV lineage.

## Abstract 2

TOC Summary: The epidemiologic pattern and limited laboratory testing indicate that this virus lineage might account for most of these illnesses.

Powassan virus is a positive-sense RNA virus that belongs to the tick-borne encephalitis group of flaviviruses (*1*). The first recognized case of Powassan encephalitis in North America was from Canada in 1958 (*2*); the first case in Russia was from the Primorsky Krai region in 1972 (*3*). Powassan virus comprises 2 closely related lineages: the Powassan virus prototype (POWV) lineage and the deer tick virus (DTV) lineage. POWV and DTV, which share 84% nucleotide sequence identity and 94% amino acid sequence identity (*4*), have a common ancestral origin, from which they diverged around 485 years ago (*5*). Each lineage has separate tick vectors and reservoir hosts in North America (*5*). POWV is maintained in an enzootic cycle between *Ixodes cookei* as the tick vector and the groundhog (*Marmota momax*) and striped skunk (*Mephitis mephitis*) as the principal reservoir hosts (*6*). DTV is believed to be maintained between *I. scapularis* ticks and the white-footed mouse (*Peromyscus leucopus*) (*7*,*8*). 

DTV can be accurately differentiated from POWV only by genetic sequence analysis. Four cases of proven DTV encephalitis have been reported: 1 from Ontario, Canada (*4*,*9*); 2 from New York (*10*,*11*); and 1 from Minnesota (*12*). For clarity, in this article, we will use the term POWV/DTV to designate infection with Powassan virus of undetermined lineage. We present a detailed description of the clinical signs and symptoms, laboratory diagnosis, and outcome of the 14 cases of POWV/DTV encephalitis diagnosed during 2004–2012 in New York. We also provide a review of the literature for epidemiologic evidence suggesting that many of these cases were caused by DTV rather than POWV.

## Methods

### Case Definition and Study Design

We conducted a retrospective review of the medical records of all POWV/DTV cases that were reported to the New York State Department of Health (NYSDOH) during 2004–2012. During this period, the NYSDOH initiated the use of diagnostic testing methods to detect this virus for all patients for whom arboviral testing was requested. We also reviewed published reports of 6 of these cases for additional details (*10*,*11*,*13*–*15*). POWV/DTV neuroinvasive infection was defined by using the 2011 United States surveillance case definition (*16*), which includes clinical criteria (fever of >38°C with any peripheral or central nervous system dysfunction documented by a physician and the absence of another more likely explanation) and >1 of the following:

- Isolation of POWV/DTV from, or detection of specific viral antigen or nucleic acid in, tissue, blood, cerebrospinal fluid (CSF), or another body fluid;
- A >4-fold change in POWV/DTV-specific quantitative antibody titers in paired serum samples;
- POWV/DTV-specific IgM in serum with confirmatory POWV/DTV-specific neutralizing antibodies in the same or a later serum specimen (POWV/DTV neutralizing antibody was considered specific if the titer was >4-fold higher than the corresponding neutralizing antibody titer to West Nile virus [WNV] or Saint Louis encephalitis virus [SLEV]);
- POWV/DTV-specific IgM antibodies in CSF and a negative result for other IgM antibodies in CSF for arboviruses endemic to the region where exposure occurred.

Demographic data were summarized by using descriptive statistics, mean and SD for continuous variables, and numbers and percentages for categorical variables. The study was approved by the Institutional Review Board at New York Medical College.

### Laboratory Analyses

Serologic testing for POWV/DTV, performed at the NYSDOH Wadsworth Center in Albany, included a microsphere immunoassay to detect IgM and, separately, total antibodies (IgG + IgA + IgM) against recombinant DTV envelope protein in serum. The microsphere immunoassay also was used to detect IgM antibodies against recombinant DTV envelope protein in CSF. Recombinant DTV envelope protein was produced from the DTV-Ipswich strain, as described (*17*,*18*). Results were assessed as the ratio of the median fluorescence intensity (MFI) for 100 beads that reacted with the patient’s serum to the MFI of beads that reacted with a negative control serum specimen. The cutoff for a positive result was a value of 3 SDs above the mean MFI result based on a panel of serum specimens from healthy subjects.

Serum and CSF samples from 2 patients were also tested for POWV/DTV at the Centers for Disease Control and Prevention diagnostic and reference laboratory (Arboviral Diseases Branch, Fort Collins, CO, USA) by using an IgM antibody capture ELISA (MAC-ELISA) and IgG ELISA against POWV envelope protein (LB strain, Canada, 1958). A plaque reduction neutralization test (PRNT) against POWV (LB strain, Canada, 1958) (*2*) was performed at the Wadsworth Center and/or the Arboviral Diseases Branch by using BHK-21 cells. The antibody titer reported is the reciprocal of the dilution of serum that inhibited 90% of the test virus inoculum. PCR for POWV/DTV and genetic sequence analysis were done at the Wadsworth Center, as described (*10*,*11*). Serum samples submitted to the NYSDOH for arboviral screening were also tested for antibodies to WNV by using a MAC-ELISA and for antibodies to SLEV by using an indirect immunofluorescence assay; if these assays were positive, specific WNV and SLEV neutralizing antibodies were measured by using PRNT.

## Results

### Demographic Characteristics

**Figure 1:**
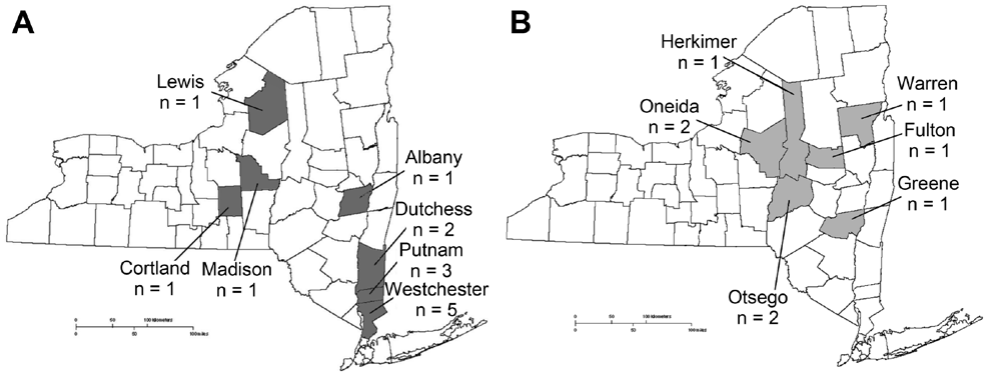
Cases of Powassan/deer tick virus encephalitis, by county, New York, USA, for 2004–2012 (A) and 1958–2003 (B) (*19*). A total of 14 cases occurred during 2004–2012 and 9 cases during 1958–2003. One additional case from 1958–2003 is not shown because the patient had lived in and traveled through multiple counties in the 6 weeks before illness onset (*20*).

**Figure 2:**
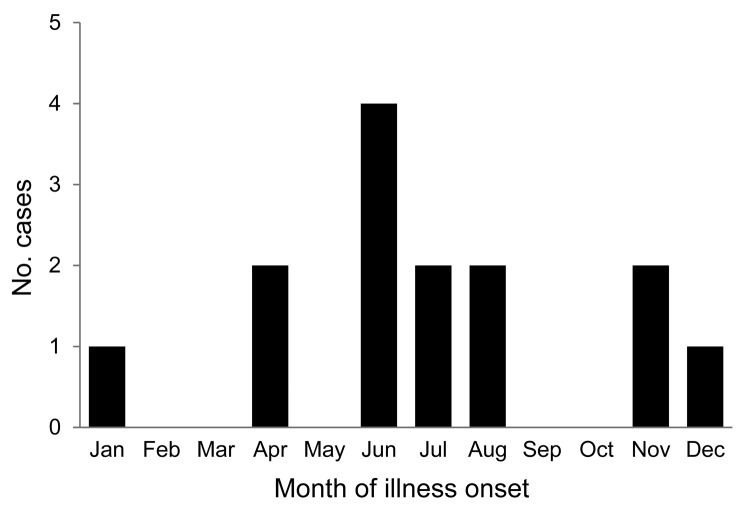
Cases of Powassan/deer tick virus encephalitis, by month of illness onset, New York, USA, 2004–2012.

Fourteen cases of POWV/DTV encephalitis were identified in New York during 2004–2012; geographic locations by case-patient county of residence are shown in Figure 1. Ten (72%) case-patients were from Westchester, Putnam, or Dutchess Counties, which are located in the Lower Hudson Valley (LHV), a highly Lyme disease–endemic region. Three (21%) patients were <10 years of age; 10 (72%) were >60 years of age. The male-to-female ratio was 3:1. Eight (57%) patients had symptom onset in June, July, or August (Figure 2). Ten (72%) patients lived in a wooded area or reported outdoor exposure, and 8 (57%) had a pet (mainly a dog or cat). A tick bite was reported for 5 (36%) case-patients before illness onset, but only 3 remembered the exact date; incubation times from the tick bite to the onset of symptoms in these patients were 9, 11, and 32 days.

All case-patients were hospitalized. The mean ± SD time from onset of symptoms to hospitalization for 13 patients was 3.5 ± 1.9 (range 1–6) days. For the remaining patient, time from onset of symptoms to hospitalization was reported to be 2–3 weeks. Seven case-patients had been prescribed an oral antimicrobial drug (amoxicillin or doxycycline) within the few days preceding hospital admission: 4 for a febrile illness with a nonspecific macular or papular rash, 2 for erythema migrans (both lived in the LHV), and 1 for headaches and malaise following a tick bite.

### Clinical and Radiographic Data

Fever (100%), generalized weakness (86%), and lethargy (72%) were the most commonly reported signs and symptoms (Table 1); 11 patients (79%) had a temperature >39°C at the time of hospitalization. Results of computed tomography scans of the brain were negative for acute findings for 13 (93%) case-patients (whether intravenous contrast was given is unknown). Results of magnetic resonance imaging (MRI) (with and without contrast for 4 patients, without contrast for 2, unknown for 8) showed acute abnormalities in 13 (93%) case-patients (mean ± SD time to MRI after hospitalization 2.9 ± 2.3 [range 1–10] days). Scattered T2-hyperintense foci predominantly affecting the gray matter and acute ischemic changes were the most common abnormalities found (Table 2). Among the 9 patients for whom electroencephalography (EEG) data were available, epileptic waveforms were documented for 3 (33%) and diffuse or severe slowing for 7 (78%) (mean ± SD time to EEG after hospitalization 9.3 ± 9.5 [range 2–30] days).

**Table 1:** Clinical signs and symptoms of 14 hospitalized patients with Powassan/deer tick virus encephalitis, New York, USA, 2004–2012

Sign or symptom	No. (%) patients
Fever	14 (100)
Generalized weakness	12 (86)
Lethargy	10 (72)
Confusion	8 (57)
Seizure	6 (43)
Headache	6 (43)
Rash	6 (43)
Nonneurologic symptoms other than rash*	6 (43)
Vomiting	5 (36)
Focal deficit	5 (36)
Neck stiffness	5 (36)
Aphasia	3 (21)
Tremor	2 (14)
Dizziness	2 (14)
Dysarthria	1 (7)
Balance disturbances	1 (7)
Myoclonus	1 (7)

*Other symptoms included dyspnea in 2 (14%), abdominal pain in 3 (21%), diarrhea in 1 (7%), dysuria in 1 (7%), body aches in 3 (21%), and rhinorrhea in 1 (7%)

**Table 2:** Brain areas affected in 13 hospitalized patients with Powassan/deer tick virus encephalitis, New York, USA, 2004–2012

Areas affected	No. (%) patients
Regions	
Cerebral cortex	7 (54)
Basal ganglia	7 (54)
Brain stem	4 (31)
Cerebellum	3 (23)
Thalamus	3 (23)
Meninges	2 (15)
Sides	
Left	9 (69)
Right	2 (15)
Bilateral	2 (15)

### Laboratory Findings

The mean ± SD hemoglobin level for all case-patients was 12.3 ± 1.62 g/dL; 12 case-patients were anemic based on reference ranges for age and sex. Mean ± SD platelet count was 217 ± 86 × 10^9^/L (reference range 172–450 × 10^9^/L). With the exception of 1 patient who had chronic lymphocytic leukemia, the mean ± SD leukocyte count at admission was 9.36 ± 3.99 × 10^9^ cells/L (reference range 4.4–11.3 × 10^9^ cells/L). Aspartate and alanine aminotransferase enzyme levels were within reference ranges.

CSF analysis (mean ± SD time to initial lumbar puncture after hospitalization 3.3 ± 3.9 [range 0–15] days) showed a mean ± SD leukocyte count of 84 ± 88 (range 0–263) cells/mm^3^. Of the 13 patients who had CSF pleocytosis, 12 had a predominance of lymphocytes; 1 patient had a CSF leukocyte count of 68 with 65% neutrophils. Although CSF in 1 patient initially showed no leukocytes, repeat CSF testing 2 days later showed 891 leukocytes with 93% lymphocytes, 6% monocytes, and 1% neutrophils. The mean ± SD CSF glucose and protein levels were 86 ± 43 (range 39–179) mg/dL and 76.7 ± 28 (range 52–142) mg/dL, respectively. Results of Gram stain testing and bacterial culture of CSF were negative for all patients.

For 2 case-patients who were residents of Putnam County, diagnosis was confirmed by detection of a conserved region in the POWV nonstructural protein 5 gene by real-time reverse transcription PCR. A positive result was obtained from brain tissue (from an autopsy specimen) from 1 patient, collected on day 17 after hospitalization, and from a CSF sample from the other patient, obtained on day 9 after hospitalization. Genetic sequencing analysis demonstrated that both of these patients were infected with DTV (Table 3). These 2 patients were previously reported (*10*,*11*). For 5 other case-patients for whom CSF was available for molecular testing, viral RNA was not detected by the same method.

**Table 3:** Diagnostic evaluation and outcome of POWV/DTV encephalitis, by county and date of illness, New York, USA, 2004–2012*

Category	Data by case no.													
	1	2	3	4	5	6	7	8	9	10	11	12	13†	14
Patient age, y	91	83	70	5	62	77	81	81	9	4	76	73	77	32
Date of illness	Jun 2004	Aug 2005	Jun 2007	Aug 2007	Jun 2007	Nov 2007	Nov 2007	Jul 2007	Jul 2008	Apr 2009	Jan 2009	Jun 2009	Dec 2010	Apr 2012
County	Alb	West	Cort	Lewis	Put	West	West	West	West	Dutch	Mad	Put	Put	Dutch
LHV	No	Yes	No	No	Yes	Yes	Yes	Yes	Yes	Yes	No	Yes	Yes	Yes
POWV MIA														
Acute	Pos	Neg	Pos	Pos	ND	Pos	Pos	Neg	Pos	Pos	Neg	Pos	Pos	Pos
Conv	Pos	Pos	Pos	Pos	ND	Pos	Pos	Ind	Pos	Pos	Pos	Pos	Pos	Pos
PRNT titer														
Acute	40,960	320	320	20	ND	320	1,280	<10	10	320	<10	2,560	5,120	1,280
Conv	40,960	1,280	320	80	ND	320	5,120	40	160	2,560	40	20,480	5,120	320
PCR for POWV/DTV NS5 gene	ND	ND	ND	ND	Pos (brain)	ND	ND	Neg	ND	Neg	Neg	Neg	Pos (CSF)	Neg
Lineage	U	U	U	U	DTV	U	U	U	U	U	U	U	DTV	U
Outcome	LADL	LADL	Died	LADL	Died	Died	LADL	Died	LADL‡	LADL	LADL	LADL‡	Died	LADL
WNV testing														
ELISA	IgM–, IgG+	Neg	Neg	IgM–, IgG+	ND	Neg	Neg	Neg	Neg	Neg	Neg	Neg	Neg	Neg
PRNT	Neg	Neg	NA	Neg	NA	Neg	NA	NA	NA	Neg	NA	NA	Neg	Neg
SLEV PRNT														
Acute	Neg	10	Neg	NA	NA	Neg	NA	NA	NA	Neg	NA	NA	Neg	Neg
Conv	Neg	80	Neg	NA	NA	Neg	NA	NA	NA	Neg	NA	NA	Neg	Neg

*All tests were done on serum samples except as indicated. POWV, Powassan virus prototype strain; DTV, deer tick virus; Alb, Albany; West, Westchester; Cort, Cortland; Put, Putnam; Dutch, Dutchess; Mad, Madison; LHV, Lower Hudson Valley, MIA, microsphere immunoassay; ND, not done; Pos, positive; Neg, negative; Conv, convalescent; Ind, indeterminate; PRNT, plaque reduction neutralization test; NS5, nonstructural protein 5 gene; CSF, cerebrospinal fluid; U, Unknown; LADL, limited activity of daily living; WNV, West Nile virus; SLEV, Saint Louis encephalitis virus; NA, not available. †Acute-phase CSF POWV IgM+ by MIA performed at Wadsworth Laboratory, New York State Department of Health; convalescent-phase CSF POWV IgM+ and IgG+ by ELISA performed at the Centers for Disease Control and Prevention (PRNT titer 8,192). ‡Imbalance.

For 12 case-patients, the diagnosis of POWV/DTV infection was made by serologic testing alone. Of these, 11 had a positive test for IgM in serum to recombinant DTV envelope protein and evidence of neutralizing antibodies against POWV; case-patient 8 had a 4-fold increase in neutralizing antibodies against POWV between acute and convalescent serum samples, without detectable IgM. In total, 8 (67%) of the 12 patients had a >4-fold increase in POWV PRNT titers between acute and convalescent serum samples (Table 3). Four patients had weak-positive test results for serum antibodies against SLEV, but serum PRNT titers for this virus were negative for 3 patients and at least 4-fold lower than the PRNT titer for POWV/DTV in 1 patient. The 2 patients with erythema migrans had detectable antibodies to *Borrelia burgdorferi*, the causative agent of Lyme disease, by standard 2-tier testing.

Histologic examination of brain tissue was available for 2 patients, 1 from a brain biopsy and 1 from a specimen obtained at autopsy. In both cases, hematoxylin and eosin stained sections revealed a reactive gliosis, increased numbers of microglial cells, and necrotizing inflammation with a lymphocytic infiltrate, predominantly affecting the gray matter, consistent with acute meningoencephalitis. For 1 of the patients, who had confirmed DTV infection by molecular analysis, a detailed histopathologic analysis showed lymphocytic infiltrates, which in the leptomeninges and perivascular spaces contained predominantly CD4+ helper T cells and in the brain parenchyma predominantly CD8+ cytotoxic T cells (*10*).

Molecular testing for 12 viruses besides POWV/DTV, including enteroviruses, herpesviruses, and arboviruses, was performed on CSF to investigate other potential viral causes of encephalitis for the 14 patients (*21*). CSF real-time PCR testing showed Epstein-Barr virus infection (EBV) at 36.2 cycle threshold (C_t_) (weak signal) and human herpesvirus 6 (HHV-6) at 29.62 C_t_ in case-patient 3 and herpes simplex virus 1 at 37.25 C_t_ (weak signal) in case-patient 11.

### Hospital Course

All patients received intravenous antimicrobial drugs during the first week of hospitalization (ceftriaxone [92%], vancomycin [67%], ampicillin [42%], and acyclovir [86%]). Case-patient 13 received oral ribavirin and subcutaneous pegylated interferon α for 2 weeks (*11*). Of the 14 patients, 5 (36%) received intravenous corticosteroids during illness (dexamethasone 0.5–1.0 mg/kg/d for 10 days or methylprednisolone 500–1,000 mg/d for 3–5 days). Time of initiation of systemic corticosteroid therapy ranged from 1 to 45 days after hospitalization.

Twelve (86%) patients were admitted to the intensive care unit (ICU) during hospitalization (mean ± SD time from hospitalization to ICU admission [n = 11] 2 ± 1.6 [range 1–6] days). Seven patients (50%) required endotracheal intubation and mechanical ventilation (mean ± SD time from hospitalization to intubation 4.6 ± 1.8 [range 3–7] days), and 5 (36%) required tracheostomy and gastric feeding tube placement.

### Outcomes

Patients were followed for a mean ± SD time of 66 ± 67 (range 10–240) days after the date of hospitalization. The mean ± SD hospital stay was 33 ± 22 (range 9–90) days. For patients admitted to the ICU for whom length of stay was available (n = 10), the mean ± SD ICU stay was 22 ± 17 (range 5–58) days. 

Five (36%) patients died. One patient died in the hospital after withdrawal of life support according to the family’s wishes; 4 died after discharge from the hospital (mean ± SD time to death from the date of hospitalization, 116 ± 94 [range 13–240] days). Although none of the postdischarge deaths could be directly attributed to POWV/DTV infection, they did appear to be related to the severely impaired health status caused by the infection. All patients who died were >60 years of age. Both patients who had DTV infection confirmed by genetic sequencing died. All 5 patients treated with corticosteroids survived; 5 (71%) of the 7 patients who did not receive corticosteroids died. (Information on corticosteroid use was unavailable for 2 patients, both of whom survived.) All of the patients who were discharged from the hospital (n = 13) had neurologic deficits at the time of discharge (Table 4).

**Table 4:** Neurologic deficits at the time of discharge in hospitalized patients with Powassan/deer tick virus encephalitis, New York, USA, 2004–2012*

Neurologic deficit	No. (%) patients
Significant limitation in ADL, n = 13	11 (85)
Cognitive deficit, n = 11	6 (55)
Bed bound, n = 13	7 (54)
Focal deficit, n = 10	4 (40)
Quadriplegia, n = 9	3 (33)
Ventilator dependence, n = 11	3 (27)
Aphasia, n = 11	3 (27)
Imbalance, n = 11	2 (18)
Headache, n = 11	2 (18)
Ophthalmoplegia, n = 9	1 (11)

*n, no. patients evaluable; ADL, activities of daily living.

## Discussion

Since the initial recognition of POWV/DTV as a cause of viral encephalitis in 1958, only ≈80 cases of POWV/DTV encephalitis have been reported (*6*,*9*–*15*,*22*–*26*). In this study, we describe 14 patients with POWV/DTV encephalitis in New York during 2004–2012. Ten of the 14 cases occurred in residents of 3 counties in the LHV, a highly Lyme disease–endemic area. Although 9 cases of POWV/DTV infections were reported in New York before 2004 (*19*,*20*,*22*), none of those cases occurred in patients from the LHV (Figure 1).

Distinguishing between POWV and DTV infection provides epidemiologically relevant information from a public health perspective. Two patients had confirmation of DTV lineage by genetic sequencing; both lived in the LHV. For several reasons, we suspect that the other 8 patients from this region were also infected with DTV. Bites by *I. cookei* ticks are rare in the LHV, whereas *I. scapularis* tick bites are common. Among 126 ticks collected from tick bite victims and submitted to the Westchester County Health Department in 1985, a total of 96 (76.2%) were identified as *I. scapularis*; none was *I. cookei* (*27*). In addition, of the 5,738 ticks submitted to the NYSDOH from persons in the LHV with tick bites during 2004–2011, only 52 (1.2%) were identified as *I. cookei*; 4,225 (72%) were identified as *I. scapularis* (NYSDOH, unpub. data). DTV is also well documented in *I. scapularis* ticks from this region and from multiple other geographic areas, whereas numerous studies have not detected the prototype POWV lineage in *I. scapularis* ticks (*7*,*8*,*28*–*32*), even though these ticks are vector competent for POWV (*33*). In a field investigation conducted during 2007–2012 and involving >13,500 nymphal and adult ticks of 7 species (including >6,100 *I. scapularis* ticks) collected throughout the LHV, DTV, but not prototype POWV, was detected exclusively in *I. scapularis* ticks, and adult ticks from the LHV had infection rates of up to 6% (*34*). 

Further lending support to our hypothesis, of the 8 POWV/DTV encephalitis case-patients from the LHV for whom virus sequence data were not available, 2 (25%) had evidence of Lyme disease: erythema migrans in conjunction with seropositivity for antibodies to *B. burgdorferi*. *B. burgdorferi* is not transmitted by *I. cookei* ticks (*35*). In a study of adult *I. scapularis* ticks collected in 2008 from Westchester County, 2 (29%) of 7 ticks infected with DTV were co-infected with *B. burgdorferi*; a third tick was co-infected with *Anaplasma phagocytophilum* (*28*). The frequency of co-infection in human cases may be lower than the co-infection rates in ticks because DTV can be transmitted within 15 minutes after onset of tick feeding, as compared with *B. burgdorferi*, which typically takes at least 48 hours (*29*). This short time required for transmission of DTV can also help explain why up to 50% of the cases from the LHV occurred during spring and fall, when adult *I. scapularis* ticks are more active; these ticks are less likely to go unnoticed than nymphs and thus may not remain attached long enough to transmit *B. burgdorferi*.

An accurate estimate of human deaths attributable to POWV/DTV infection remains unknown. Unlike the all-cause mortality rate of 36% that we observed, previous studies have reported fatality rates of <20% for POWV/DTV neuroinvasive infection (*9*,*13*,*22*). Differences in the length of follow-up might partly explain this difference; our 30-day fatality rate was also <20%. Several factors may have been associated with poor outcomes in this case series. Among the 5 patients who died, 4 were from the LHV, 2 with genetic sequencing–confirmed DTV infection and 1 with Lyme disease co-infection. This finding indicates a notable proportion of probable DTV-related deaths in our cohort. The 5 patients who died were >60 years of age, and 3 had brain stem or cerebellar involvement on MRI. In contrast, only 1 (11%) of the 9 survivors had brain stem or cerebellar involvement on MRI. Thus, age >60 years and involvement of the rhombencephalon may be poor prognostic indicators for POWV/DTV encephalitis.

No effective therapy is available for POWV/DTV encephalitis; current guidelines recommend supportive therapy (*36*). The role of antiviral therapy remains unclear. The case-patient in our series who was treated with ribavirin plus interferon 3 weeks into his illness (which may be late) did not improve and eventually died (*11*). In contrast, all of the patients who received corticosteroids during their illness survived; therefore, future studies are needed to evaluate the potential role of systemic corticosteroids in the treatment of POWV/DTV encephalitis.

Although all of the case-patients in this study tested positive for POWV/DTV, HHV-6 DNA (29.62 C_t_) and low levels of EBV DNA (36.2 C_t_) were detected in the CSF of 1 patient. A high rate of detection of HHV-6 in CSF from healthy adults has been described; therefore, the clinical significance of detection of HHV-6 in CSF in immunocompetent hosts remains unclear (*36*). EBV is also occasionally detected in CSF at low levels and may be of no clinical significance (*37*). Similarly, PCR for HSV had a low-positive result (37.25 C_t_) in the CSF of a patient who did not have the characteristic temporal lobe involvement of HSV encephalitis on EEG or on MRI (*38*) and did not respond to acyclovir therapy. The diagnosis of POWV/DTV encephalitis in this patient was made on the basis of a positive test result for serum IgM against POWV/DTV and a 4-fold increase in specific neutralizing antibodies against POWV. Thus, if this patient were co-infected with HSV and POWV/DTV, the latter may have contributed to the disease severity, but to what extent remains unclear. If, in fact, the correct diagnosis was HSV encephalitis alone, then the proportion of POWV/DTV encephalitis cases from the LHV was actually higher than we have reported, since this patient was from another part of New York.

In conclusion, we describe 14 cases of POWV/DTV encephalitis from New York diagnosed during 2004–2012. Ten (72%) of the case-patients were from the LHV, a highly endemic area for Lyme disease. We suspect that the cases occurring in the LHV may have been caused by DTV. Given the high rate of severe illness and death associated with these infections and the evolving epidemiology, molecular analysis is essential in the evaluation of POWV/DTV infections.
